# MicroRNA-650 Regulates the Pathogenesis of Alzheimer’s Disease Through Targeting Cyclin-Dependent Kinase 5

**DOI:** 10.1007/s12035-023-03224-y

**Published:** 2023-01-19

**Authors:** Li Lin, Xiaodong Liu, Xuejun Cheng, Yujing Li, Marla Gearing, Allan Levey, Xiaoli Huang, Ying Li, Peng Jin, Xuekun Li

**Affiliations:** 1 Guangdong-Hongkong-Macau Institute of CNS Regeneration, Guangdong Key Laboratory of Nonhuman Primate Models of Human Diseases, Key Laboratory of CNS Regeneration (Ministry of Education), Jinan University, Guangzhou, 510632 China; 2 The Children’s Hospital, School of Medicine, Zhejiang University, Hangzhou, 310003 China; 3 The Institute of Translational Medicine, School of Medicine, Zhejiang University, Hangzhou, 310003 China; 4 Department of Human Genetics, Emory University School of Medicine, Atlanta, GA 30322 USA; 5 Center for Neurodegenerative Disease, Emory University School of Medicine, Atlanta, GA 30322 USA

**Keywords:** Alzheimer’s disease, Cyclin-Dependent Kinase 5 (CDK5), microRNA (miRNA), miR-650

## Abstract

Alzheimer’s disease (AD) pathogenesis feature progressive neurodegeneration, amyloid-β plaque formation, and neurofibrillary tangles. Ample evidence has indicated the involvement of epigenetic pathways in AD pathogenesis. Here, we show that the expression of microRNA 650 (miR-650) is altered in brains from AD patients. Furthermore, we found that the processing of primary miR-650 to mature miR-650 is misregulated. Bioinformatic analysis predicted that miR-650 targets the expression of three AD-associated components: Apolipoprotein E (APOE), Presenilin 1 (PSEN1), and Cyclin-Dependent Kinase 5 (CDK5), and we have experimentally confirmed that miR-650 is able to significantly reduce the expression of APOE, PSEN1, and CDK5 in vitro. Importantly, the overexpression of miR-650 was further shown to significantly alter the CDK5 level and ameliorate AD pathologies in APP-PSEN1 transgenic mice. Overall, our results indicate that miR-650 influences AD pathogenesis through regulation of CDK5.

**Supplementary Information:**

The online version contains supplementary material available at 10.1007/s12035-023-03224-y.

## Introduction

Alzheimer’s disease (AD) is the most common form of neurodegenerative diseases, and the leading cause of dementia. Although the etiology of AD is still rather vague, multiple neuropathological hallmarks of AD have been characterized, including insoluble, extracellular aggregates of amyloid-β peptides (Aβ), named amyloid plaques, and intracellular accumulations of hyperphosphorylated microtubule-associated protein tau, named neurofibrillary tangles (NFTs), as well as neuronal loss [1–4]. At the molecular level, tremendous efforts have been made to dissect various pathways and components underlying the pathogenesis of neurodegenerative lesions observed in AD. The processing of amyloid precursor protein (APP) to Aβ is catalyzed by β-secretase and γ-secretase complex sequentially, and the resultant cleaved short forms of Aβ can then self-aggregate to form amyloid plaques. It is widely accepted that the amyloidogenesis results in the activation of various kinases, leading to the neurotoxic phosphorylation of tau [5]. Nonetheless, the molecular basis, especially at the early onset of AD, is still unclear.

The interactions between protein kinase and phosphatase activities, via switching on and off of protein phosphorylation, regulate numerous cellular processes. Indeed, abnormally phosphorylated proteins have been conclusively documented in various diseases, including degenerative diseases in the brain [6–9]. In particular, several kinases that regulate the production of Aβ peptide, as well as tau phosphorylation, were altered in AD patients [10–16]. For instance, inhibition of CDK5 appears to protect β-amyloid-induced neuronal cell death; treatment with therapeutic concentration of a glycogen synthase kinase inhibitor blocked the generation of β-amyloid peptides. However, little is known about the regulating mechanisms of the related kinase expression in the context of AD pathogenesis.

As one component of the epigenetic landscape, microRNAs (miRNAs) are small non-coding RNAs with a length of ~ 22 nucleotides (nt). The biogenesis of miRNA is a multi-step process [17–20]. Transcribed primary miRNAs (pri-miRNAs) are processed by Drosha in the nucleus, and the resulting precursor miRNAs (pre-miRNAs) are exported to the cytoplasm where they are further processed by Dicer to generate mature miRNAs. The produced miRNAs regulate gene expression through binding of the 3′-untranslated regions (3′-UTR) of target mRNAs. Previous studies have shown that miRNAs play essential functions in a diversity of biological processes including cancer, development, aging, and disease progression [21–26]. In neuronal systems, previous studies have demonstrated that miRNAs regulate neuronal development and synaptic plasticity, toxic protein accumulation, and cell death and survival, suggesting their involvement in neuronal degenerative diseases [22, 25, 27]. The aberrant miRNA levels have been indicated in the pathogenesis of neurodegenerative diseases including Parkinson’s disease and Alzheimer’s disease [28–35]. Nonetheless, how the deregulated miRNAs might contribute to disease pathogenesis is still not fully understood.

To gain more insight into the function of miRNAs in the pathogenesis of AD, we profiled the miRNA expression in the brain of normal and AD patients and uncovered several miRNAs that showed a significant alteration among AD cases. Using bioinformatic analyses, we determined that miR-650, one of these altered miRNAs, was likely to bind to APOE, PSEN1, and CDK5, genes involved in AD pathogenesis. We further found that the process from primary/precursor to the mature form of miR-650 was dysregulated in AD cases. Importantly, we discovered that miR-650 can regulate APOE, PSEN1, and CDK5 expression levels in vitro*,* and we went on to determine that miR-650 could also regulate CDK5 levels in vivo using APP/PSEN1 mice, a conventional model for AD. Over-expression of miR-650 could also ameliorate neuronal deficits in these AD mice as evidenced by a decrease in the number of plaques and decreased Aβ levels. Our studies indicate that miR-650-CDK5 axis plays a regulatory role in the pathogenesis of AD.

## Materials and Methods

### Patient Information

Brain tissues of control and AD patients were obtained from archival human brain specimens of the Emory ADRC Neuropathology Core, using appropriate IRB protocols. Neuropathologic examination of every subject was recorded, and patients were diagnosed according to the National Institute on Aging/Alzheimer’s Association (NIA/AA) criteria [36]. Criteria for inclusion are the following: (1) post-mortem interval <  = 8 h; (2) patient aged 65 and older; (3) no complicating agonal condition or underlying CNS disease, including stroke, neoplasms, or features of non-AD dementia (cortical Lewy bodies); and (4) extensive premortem cognitive testing (followed at least 3 years, with a battery of tests administered within one year of death). In order to provide adequate inter- and intra-case controls, tissues must be sampled from different cerebral cortical areas. The inclusion of an age-matched cohort with no cognitive impairment as a control group for comparison with the AD cases is also essential. Superior frontal (Brodmann area 9) and paracalcarine cortex (Brodmann area 17) from both controls and patients were collected by the Emory ADRC Neuropathology Core and used for RNA isolation. The information of patients and healthy controls was summarized in Table 1.

**Table 1:** Information of patients and healthy controls

Sample	PMI	msex	braaksc	age_death	educ	race
AD-1	4.416667	M	5	88.0328542	18	Caucasian
AD-2	5.333333	M	5	83.1362081	12	Caucasian
AD-3	4.833333	M	5	83.4976044	16	Caucasian
AD-4	18.66667	F	5	84.8747433	17	Caucasian
AD-5	5.833333	M	5	84.7556468	18	Caucasian
AD-6	21.75	F	5	85.615332	16	Caucasian
AD-7	6.166667	F	5	82.3258042	18	Caucasian
AD-8	12.38333	M	5	85.8576318	16	Caucasian
AD-9	8.75	F	5	91.4195756	21	Caucasian
AD-10	5.75	M	5	84.752909	16	Caucasian
AD-11	18.5	F	5	84.3039014	21	Caucasian
AD-12	6.5	F	5	88.4462697	15	Caucasian
AD-13	15.16667	F	5	84.9883641	19	Caucasian
AD-14	8.2	M	5	90.5954826	22	Caucasian
AD-15	12.88333	M	5	86.8117728	12	Caucasian
AD-16	10.58333	F	5	84.440794	18	Caucasian
AD-17	8.666667	F	5	86.9322382	24	Caucasian
AD-18	6.083333	M	5	89.0321698	11	Caucasian
CTRL-1	7.75	M	0	89.0869268	12	Caucasian
CTRL-2	1.75	F	1	81.7344285	19	Caucasian
CTRL-3	18.21667	F	0	79.1156742	18	Caucasian
CTRL-4	29	M	1	86.2149213	24	Caucasian
CTRL-5	22.33333	M	1	80.1806982	12	Caucasian
CTRL-6	5.916667	F	0	85.9945243	12	Caucasian
CTRL-7	4.75	M	0	84.6009583	21	Caucasian
CTRL-8	7.5	F	0	87.2854209	18	Caucasian
CTRL-9	31	M	0	87.9616701	13	Caucasian
CTRL-10	10.08333	M	0	84.3196441	20	Caucasian
CTRL-11	3	F	1	85.8466804	14	Caucasian
CTRL-12	7.5	F	0	82.4818617	15	Caucasian
CTRL-13	4.083333	M	1	81.6249144	26	Caucasian
CTRL-14	7.5	F	1	85.4332649	18	Caucasian
CTRL-15	7.016667	M	0	84.4503765	14	Caucasian
CTRL-16	4.466667	M	0	82.5968515	15	Caucasian

### APP/PSEN1 Double Transgenic Mice

Age-matched male wild-type (WT) and APP/PSEN1 double transgenic littermate mice were used in this study. APP/PSEN1 double transgenic mice expressing a chimeric mouse/human APP with the Swedish mutation and a human PSEN1 Δexon 9 mutation [37, 38] were obtained from The Jackson Laboratory. Mice were maintained at ambient temperature (22–24 °C) on a 12:12 light/dark cycle with free access to food and water. All animal procedures were performed according to protocols approved by Emory University Institutional Animal Care and Use Committee.

### Relative Quantification of Mature miRNAs by TaqMan Assay

Profiling of mature miRNA expression was performed using TaqMan miRNA assays (Applied Biosystems) as described previously [39]. In brief, 48 reverse transcription primers were used in 20-µl reactions consisting of: 20 ng total RNA, 1 × TaqMan miRNA reverse transcription primer pool, 0.5 mM of each deoxyribonucleotide triphosphate (dNTP), 10.0 U/µl MultiScribe (Applied Biosystems) reverse transcription, 1 × reverse transcription buffer, 0.25 U/µl RNase inhibitor, and nuclease-free water. The reactions were incubated at 16 °C for 30 min, 42 °C for 30 min, and 85 °C for 5 min. Reactions were diluted 1:10 with nuclease-free water for use in the TaqMan real-time PCR reactions. Individual TaqMan miRNA real-time PCR reactions for profiling experiments were performed on a PCR system (7900HT SDS; Applied Biosystems) in a 384-well format running control and AD samples in parallel for each cDNA pool generated in the reverse transcription step. PCR reactions were performed in triplicate for each sample and each miRNA. The 10-µl reactions consisted of 1 × TaqMan Universal Master Mix, No AmpErase UNG, 1 × TaqMan miRNA assay mix, 0.8 µl of 1:10 diluted cDNA, and nuclease-free water. All TaqMan PCR reactions were prepared and aliquoted using a custom method on an automated pipette (Biomek FX; Beckman Coulter). PCR reaction conditions were run according to the standard protocol without the 50 °C incubation using version 2.3 of the SDS software, with reactions incubated at 95 °C for 10 min, followed by 40 cycles of 95 °C for 15 s, and 60 °C for 1 min.

For the reverse transcription of specific miRNA, the 15-μl reverse transcription reaction consisted of 1 μg of total RNA, 1 × of specific RT primer, 5 U MultiScribe Reverse transcription, and 0.5 mM of dNTPs mixture, 1 × reverse transcription buffer, 4 U RNase inhibitor, and nuclease-free water. Reverse transcription reactions were performed as followings: 16 °C for 30 min, 42 °C for 30 min, and 85 °C for 5 min for the TaqMan assay of individual miRNAs.

Primary/precursor miR-650 was detected by polyadenylating total RNA using SuperScript III oligo-dT reverse transcription (Invitrogen) to generate first-strand cDNA and real-time PCR targeting the pri-/pre-miR-650. Polyadenylation and reverse transcription were performed according to the manufacturer’s instructions using the Ncode miRNA First-Strand cDNA Synthesis kit (Invitrogen), with 1 µg of total RNA isolated with TRIZOL as input. Relative quantification was performed by real-time PCR using 1:10 diluted cDNA.

### Plasmid Construction, Co-transfection, and Luciferase Assay

To construct the 3′-UTR-luciferase vectors, the 3′-UTR sequence of APOE, PSEN1, and CDK5 mRNA were PCR amplified from 293FT cell cDNA. The following primers were used: APOE: 3′-UTR–F: TCAGGAGCTCACGCCGAAGCCTGCAGCCATG, 3′-UTR–R: TCAGTCTAGATGCGTGAAACTTGGTGAATCT; PSEN1: 3′-UTR–F: TCAGGAGCT CC ATATTTGCGGTTAGAATCCCATG, 3′-UTR–R: TCAGTCTAGATGCTTCAACAGCCA TTTTACTCTT; and CDK5:3′-UTR–F: TCAGGAGCTCGCCCCGGGACCCCCGGCCT CCAGG, 3′-UTR–R: TCAGTCTAGAGACTGTGGGAAAG GAGCCAATTTA. The PCR products were each cloned into a pIS2 vector (Addgene) at SacI and XbaI sites.

To construct the mutant plasmids, the miR-650 target site in the 3′-UTRs of APOE, PSEN1, and CDK5 were deleted, using the QuikChange Multi Site-Directed Mutagenesis kit (Agilent Technologies, Cat# 200514). The following primers were used for mutagenesis: mutant mutant APOE: CGCTGCAGGCTGCGCCGGGGTGGCGTGGGG; PSEN1: GGGAGAGA AGAATGAGGACAGTCTGGGGCAAGTGAGCGTACA; CDK5: TCTAGAGACTGT GGGAAATTTATGAAATTAAATAAAGTCCAC. The mutant plasmids were verified by sequencing.

For the transfection, pIS2-3′UTR (0.4 μg) and pIS0 (0.4 μg) were co-transfected with miR-650 duplex at a final concentration of 2.5 nM into 293FT cells with Lipofectamine 2000 (Invitrogen, Cat#11668019) in 24-well plate. pIS0 vector (Addgene) was used for the internal control. The dual luciferase assay was performed according to the manufacturer’s instructions (Promega, Cat# TM040) 48 h after the transfection. All assays were performed in triplicate and repeated independently three times for each co-transfection.

Human miR-650 precursor sequence was fused with a GFP tag in the N terminus and subcloned into pAAV2/9n vector (Addgene). The CMV promoter sequence was inserted into the construct to replace the original promoter in the vector. AVV-GFP-miR-650 and AAV-GFP (used as a control) was packaged and amplified by the Viral Vector Core at Emory University.

### AAV Virus In Vivo Injection

The in vivo virus grafting was performed as described previously [39]. Briefly, 1-year-old APP/PSEN1 double transgenic mice were anesthetized with 1% isoflurane, and AAV virus (3ul with titer: 8.0X10^13^ VG/ml) was injected stereotaxically into the dentate gyrus (DG) using the following coordinates relative to the bregma: anteroposterior, − (1/2) × d mm; lateral, ± 1.8 mm (if d > 1.6), or otherwise ± 1.7 mm; and ventral, − 1.9 mm (from dura). For each mouse, the AAV-GFP control virus was injected into the left DG, and the AAV-miR-650 virus was injected into the right DG. The mice were sacrificed, and hippocampi were dissected out 1 month after the virus grafting for biochemical assay.

### Western Blot Analyses

The tissues were ground in ProteoJET Mammalian Cell Lysis Reagent (Fermentas, Cat#K0301) with the presence of protease inhibitor cocktail (Roche, Cat# 11836153001). Lysate was kept on ice for 30 min. Thirty-microgram total protein were separated on SDS-PAGE gels and then transferred to PVDF membranes (Millipore). Membranes were processed following the HyGLO QuickSpray western blotting protocol (Denville). The following antibodies were used: polyclonal anti-Cdk5 (C-8) (Santa Cruz, Cat# sc-173), polyclonal anti-GFP (Invitrogen, Cat#A11122), and monoclonal anti-human GAPDH (Invitrogen, Cat# 437000). The density of each band was quantified by ImageJ software. An unpaired *t*-test was used and the data are presented as mean ± SEM.

### Immunohistochemistry

Mouse brains were fixed by perfusion with phosphate-buffered saline (PBS), followed by 4% paraformaldehyde in PBS 1 month after virus injection. The brains were post-fixed in the same fixative overnight for preparing paraffin or freezing sections (30% sucrose in 0.1 M of PBS for 2 days after post-fixation). The coronal paraffin sections were cut at 5 μm thicknesses for beta-amyloid immunohistochemical staining. The sections were then incubated in 0.3% H_2_O_2_ at 37℃, followed by incubation for 2 h in blocking buffer (TBS containing 0.3% Triton X-100 and 5% normal horse serum). Next, the sections were incubated overnight with monoclonal anti-beta-amyloid 1–42 antibody (Millipore, 1:1000) in blocking buffer and then incubated for 2 h with biotinylated horse anti-mouse IgG diluted 1:100 in blocking buffer, followed by incubation for 1 h in an avidin–biotin-peroxidase complex solution.

### Quantification of Aβ Level and Positive Plaques

Mouse hippocampi were homogenized in tissue lysate buffer (20 mM Tris–HCl (pH 7.4), 1 mM ethylenediaminetetraacetic acid, 1 mM ethylene glycol tetraacetic acid, 250 mM sucrose) supplemented with protease inhibitors (Roche). The tissue (*n* = 3 per group) homogenates were treated with diethanolamine to extract soluble β-amyloid. β-amyloid was measured using Invitrogen Aβ 40 Mouse ELISA Kit (Cat# KMB3481) according to the manufacturer’s protocol. The statistical significance was calculated using an unpaired *t*-test and the data are presented as mean ± SEM.

The stained brain sections were analyzed and photographed using a Zeiss Axioplan microscope using AxioVision 4.6 software. The distribution and quantity of Aβ-positive plaques was determined in AAV-GFP and AAV-GFP-miR-650 virus injected mice (*n* = 3 per group), and 5 sections per dentate gyrus were analyzed for plaque load. Plaque loads were quantified using ImageJ software and recorded total numbers. The numbers of plaque load were averaged from 3 mice in each group and compared. The statistical significance was calculated using an unpaired *t*-test and the data are presented as mean ± SEM.

### Immunofluorescence Staining

Mice were perfused with PBS followed by 4% paraformaldehyde 1 month after virus injection and brains were removed and dehydrated in 30% sucrose. 40 µm coronal brain sections were collected on a Leica cryostat. The immunofluorescence staining was performed as described before [40]. The primary anti-mouse MAP2 antibody (Sigma, 1:500) and the secondary antibody goat anti-mouse 568 (Invitrogen, 1:500) were applied sequentially. The slides were studied on a Zeiss microscope.

### Bioinformatic Analysis

A online program TargetScan was used to predict targets of miR-650 by searching conserved 8mer, 7mer, and 6mer sites that match the seed region of each miRNA [41] (https://www.targetscan.org/vert_80/). From all potential targets, we selected key genes that involved in the pathogenesis of AD for further analysis.

### Statistical Analysis

Statistical analysis was performed using one-way ANOVA and unpaired *t*-test. All data are presented as mean with standard error of mean (mean ± SEM).

## Results

### Significantly Increased Expression of miR-650 in the Brain of AD Patients

**Fig. 1:**
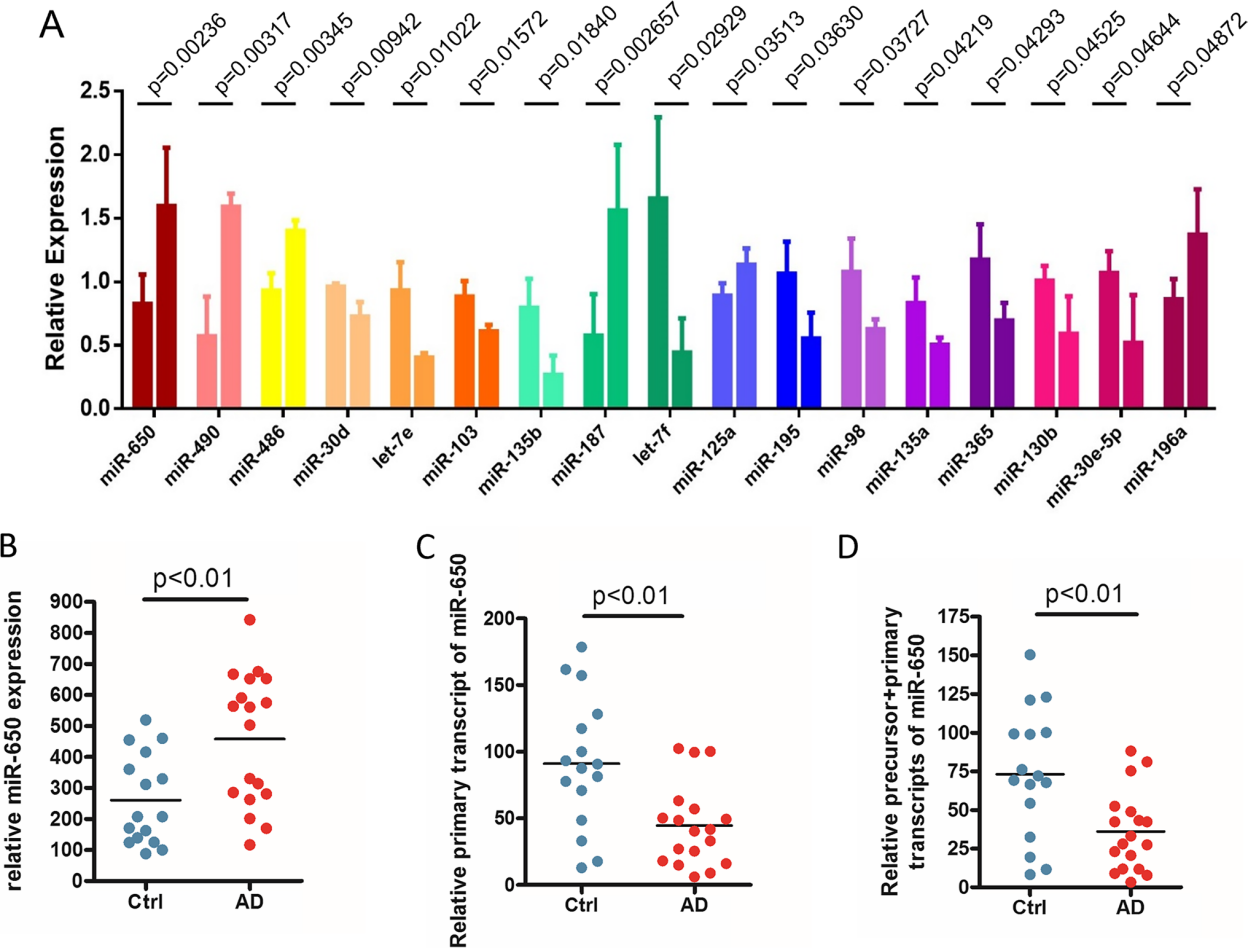
Identification of significantly altered miRNAs in brain from AD patients. **A** Relative expression levels of top 17 miRNAs from control and Alzheimer’s disease (AD) subjects. For each miRNA, left column represents the control and the right column represents the AD. Unpaired *t*-test, *p*-value showed on the top of each group. **B**–**D** Relative expression levels of matured miR-650 (**B**), primary miR-650 (**C**), and precursor and primary transcript of miR-650 (**D**) for control and AD subjects. Unpaired *t*-test, *p* < 0.01. Control, *n* = 16; AD, *n* = 18

To analyze the expression of miRNAs and reveal the specific miRNA changes in the brain samples of AD patients, a total of 204 miRNAs were analyzed using TaqMan assays (Table 2), and 17 miRNAs were significantly increased in AD samples compared to control samples (*p* < 0.05) (Fig. 1A, top 17). MiR-650 stood out as the most significantly increased one among 17 miRNAs (*p* = 0.00236, Table 2).

**Table 2:** Analysis of  the expression of 204 miRNAs in AD and controls

MicroRNAs	Control Avg	Control SD	AD Avg	AD SD	AD/control	*T* test *p*-value
miR-650	0.82625	0.2314	1.597832	0.456782	1.93384	0.00236
miR-490	0.572	0.31319323	1.5906667	0.103249	2.78089	0.00317
miR-486	0.93	0.13765416	1.4016667	0.082246	1.50717	0.00345
miR-30d	0.9605	0.02686385	0.7263333	0.113773	0.7562	0.00942
let-7e	0.933	0.22171904	0.4023333	0.036088	0.43123	0.01022
miR-103	0.88475	0.12271478	0.609	0.052	0.68833	0.01572
miR-135b	0.79575	0.22785723	0.268	0.151248	0.33679	0.0184
miR-187	0.57575	0.32838836	1.5606667	0.517912	2.71067	0.02657
let-7f	1.6545	0.64020283	0.4423333	0.271929	0.26735	0.02929
miR-125a	0.88975	0.09787875	1.134	0.129387	1.27452	0.03513
miR-195	1.06325	0.25339215	0.554	0.204007	0.52104	0.0363
miR-98	1.078	0.26242459	0.6286667	0.076134	0.58318	0.03727
miR-135a	0.83325	0.20046841	0.5023333	0.059534	0.60286	0.04219
miR-365	1.175	0.27828762	0.6953333	0.139228	0.59177	0.04293
miR-130b	1.01	0.11501884	0.5913333	0.294741	0.58548	0.04525
miR-30e-5p	1.069	0.17081179	0.5206667	0.377203	0.48706	0.04644
miR-196a	0.8645	0.15731391	1.372	0.356613	1.58704	0.04872
miR-376a	1.68875	0.50370453	0.786	0.431169	0.46543	0.05563
miR-515-3p	0.85825	0.40471255	3.8823333	2.473844	4.52355	0.05575
miR-656	0.692	0.25603776	0.2906667	0.14103	0.42004	0.06039
miR-224	1.00025	0.5383539	0.2003333	0.18859	0.20028	0.06051
miR-576	9.446	11.0110668	27.615	3.783768	2.92346	0.06697
miR-449	1.50125	0.51285955	3.3103333	1.511855	2.20505	0.07086
let-7a	1.39725	0.61932726	0.5443333	0.203166	0.38957	0.07441
miR-130a	0.81825	0.15759944	0.5873333	0.119132	0.71779	0.0889
miR-374	1.3385	0.58959789	0.5996667	0.210334	0.44801	0.09766
miR-206	1.87775	0.99398168	0.5416667	0.679253	0.28847	0.10403
miR-497	0.9925	0.204107	0.7236667	0.134374	0.72914	0.10715
miR-181c	1.7485	0.90044822	0.554	0.647755	0.31684	0.111
miR-149	0.73525	0.19350689	1.042	0.237299	1.41721	0.11684
miR-19a	1.3825	0.3889083	0.6263333	0.69074	0.45304	0.12108
miR-646	0.7005	0.29738695	1.3836667	0.670197	1.97526	0.12289
miR-126	1.4525	0.39259266	0.9796667	0.234338	0.67447	0.12677
miR-659	0.55575	0.38211026	0.137	0.076492	0.24651	0.12707
miR-191	1.46025	0.93298959	2.5243333	0.425112	1.7287	0.13061
miR-190	1.662	0.69813704	0.7356667	0.629299	0.44264	0.13069
miR-124a	0.57625	0.35487592	0.957	0.070378	1.66074	0.13343
miR-487b	0.768	0.20448798	0.5453333	0.076252	0.71007	0.13859
RNU44	0.838	0.11651895	0.674	0.130215	0.8043	0.13918
miR-518e	1.31925	0.45931353	2.4776667	1.250399	1.87809	0.14069
miR-342	0.80075	0.14757682	0.9733333	0.097439	1.21553	0.14235
miR-218	0.61675	0.35341512	1.0013333	0.151837	1.62356	0.14314
miR-489	2.64725	1.2513258	4.4346667	1.516503	1.6752	0.14677
miR-519e	0.88525	0.0997242	1.4926667	0.724159	1.68615	0.14752
miR-30b	0.8665	0.11698575	0.7326667	0.077488	0.84555	0.1497
miR-383	0.609	0.29299716	0.889	0.030116	1.45977	0.16838
miR-451	0.82375	0.15331096	0.663	0.102679	0.80486	0.18066
miR-31	0.72325	0.25669616	0.983	0.145093	1.35914	0.18113
miR-324-5p	0.905	0.19074066	1.1126667	0.152054	1.22947	0.18363
miR-9	1.67175	0.23109792	1.4576667	0.064423	0.87194	0.18734
miR-134	0.63375	0.27884569	0.903	0.15762	1.42485	0.19846
miR-197	0.9135	0.1617292	0.743	0.147285	0.81336	0.21212
miR-221	0.6455	0.30424825	0.3846667	0.094204	0.59592	0.21903
miR-132	0.58275	0.36852985	0.263	0.148091	0.45131	0.22225
miR-23a	1.071	0.34799713	0.7236667	0.291214	0.67569	0.22239
miR-320	1.23975	0.55063017	2.262	1.365459	1.82456	0.22335
let-7g	1.3835	0.59946115	0.852	0.30502	0.61583	0.22495
miR-326	0.95825	0.31978782	1.283	0.304493	1.3389	0.23337
miR-382	0.68675	0.24489641	0.9246667	0.216385	1.34644	0.24043
miR-148a	1.26825	0.51384458	0.8116667	0.346503	0.63999	0.24537
miR-432	0.66225	0.29690669	0.905	0.136635	1.36655	0.25231
let-7d	1.10825	0.40427579	0.7906667	0.146678	0.71344	0.25894
miR-143	1.12675	0.15008747	1.499	0.588161	1.33037	0.26642
miR-301	1.05925	0.27609585	0.796	0.287861	0.75148	0.27437
miR-324-3p	0.90375	0.10861975	1.1983333	0.489466	1.32596	0.28307
miR-378	0.716	0.30641584	0.4896667	0.138233	0.68389	0.29415
miR-517c	0.97275	0.79339455	1.7853333	1.077756	1.83535	0.29871
miR-16	1.23125	0.44701035	0.8386667	0.44751	0.68115	0.30239
miR-32	1.5475	0.72728926	0.914	0.728406	0.59063	0.30602
miR-331	1.10725	0.42528451	1.3896667	0.112024	1.25506	0.32249
miR-15b	1.731	0.94351859	1.0913333	0.380245	0.63046	0.32602
miR-193b	1.13625	0.41368456	0.8116667	0.374428	0.71434	0.33493
miR-10a	1.71425	1.51252601	0.7606667	0.161203	0.44373	0.33696
miR-564	1.03	1.2221132	1.8436667	0.531662	1.78997	0.33745
miR-142-5p	3.1005	2.5854955	5.3323333	3.149324	1.71983	0.3483
miR-200b	1.26325	1.37244608	0.418	0.258946	0.33089	0.35071
miR-369-5p	0.6735	0.22312104	0.8273333	0.15783	1.22841	0.35921
miR-26b	1.66675	0.96631374	1.013	0.653881	0.60777	0.3628
miR-429	1.84175	1.01138927	1.0613333	1.045662	0.57626	0.36469
miR-452	1.11275	0.53815263	0.794	0.091	0.71355	0.36685
miR-21	2.2345	1.64102194	1.254	0.475719	0.5612	0.37084
miR-133a	0.71175	0.27710212	0.9626667	0.407809	1.35253	0.37251
miR-501	1.7425	1.73180301	2.982	1.563153	1.71133	0.37484
miR-34c	3.47275	4.84518244	0.6773333	0.487501	0.19504	0.37572
miR-24	1.0665	0.29201427	0.8523333	0.28495	0.79919	0.37679
miR-211	93.6153	185.921329	285.27267	341.5866	3.04729	0.37819
miR-361	0.89075	0.14074889	0.8103333	0.02203	0.90972	0.38208
miR-20b	1.251	0.37720905	0.9646667	0.422973	0.77112	0.38742
miR-146a	1.05525	0.27157734	1.208	0.03995	1.14475	0.38854
miR-34a	0.806	0.16543075	0.9143333	0.129404	1.13441	0.39371
miR-642	5.25825	5.6674166	2.0726667	1.468069	0.39417	0.39527
miR-20a	1.397	0.62884815	0.965	0.585941	0.69077	0.39782
miR-532	1.19175	0.26010046	0.9786667	0.356573	0.8212	0.39856
miR-137	0.64725	0.33432955	0.866	0.30086	1.33797	0.41362
miR-411	0.57325	0.29545375	0.7353333	0.108103	1.28274	0.41497
miR-25	1.534	0.7735774	1.0423333	0.646687	0.67949	0.41554
miR-409-5p	0.34725	0.4627349	0.6686667	0.497826	1.92561	0.4181
miR-193a	1.502	0.90104643	0.9016667	0.89244	0.60031	0.42127
miR-107	0.51975	0.36341379	0.33	0.123467	0.63492	0.4339
miR-155	1.39	0.52869147	1.045	0.543081	0.7518	0.43661
miR-30e-3p	1.07375	0.29021185	1.258	0.291861	1.17159	0.44467
miR-95	0.70725	0.198179	0.5943333	0.144504	0.84034	0.44562
miR-485-3p	0.6755	0.27305372	0.5326667	0.133822	0.78855	0.44905
miR-129	0.5815	0.3525039	0.4076667	0.089891	0.70106	0.45158
miR-182	0.792	0.33860892	0.625	0.095896	0.78914	0.45358
miR-196b	0.634	0.32567878	0.461	0.188987	0.72713	0.454
miR-323	0.75525	0.21037645	0.6353333	0.165001	0.84122	0.45402
miR-660	1.132	0.37628801	0.912	0.32142	0.80565	0.45444
miR-335	0.7555	0.22079327	0.6273333	0.186122	0.83036	0.45566
miR-99b	1.0905	0.22756318	1.2203333	0.183699	1.11906	0.45726
miR-213	1.2905	0.40240982	1.0446667	0.407503	0.80951	0.46223
miR-106b	1.27775	0.53716067	0.9766667	0.442358	0.76436	0.46734
miR-127	0.67525	0.27947138	0.8046667	0.032593	1.19166	0.4711
miR-422b	0.859	0.51890333	1.155	0.468432	1.34459	0.47276
miR-145	1.08475	0.26885111	1.3326667	0.575006	1.22855	0.47361
miR-425-5p	0.8145	0.12883452	0.7453333	0.097295	0.91508	0.47477
miR-19b	1.40675	0.62888228	1.9103333	1.141151	1.35798	0.48305
miR-146b	0.65525	0.2513104	0.5366667	0.102002	0.81903	0.48312
let-7c	1.10075	0.37771627	0.9286667	0.10061	0.84367	0.48565
miR-328	0.88925	0.1171477	0.834	0.056241	0.93787	0.49133
miR-520g	0.61075	0.42352597	0.8993333	0.620395	1.47251	0.49324
miR-30a-5p	0.8145	0.13700973	0.913	0.220402	1.12093	0.49472
miR-29a	0.999	0.140748	1.1236667	0.323186	1.12479	0.51251
miR-34b	3.9635	5.55459464	1.6283333	1.351143	0.41083	0.51687
miR-194	1.45525	0.47683357	1.8343333	0.963557	1.26049	0.51715
miR-375	0.894	0.46039693	0.6906667	0.229692	0.77256	0.52012
miR-7	0.60725	0.36160787	0.76	0.129677	1.25154	0.52366
miR-204	1.03925	0.18479606	0.9236667	0.267395	0.88878	0.52494
miR-362	0.862	0.25527632	0.746	0.161558	0.86543	0.52533
miR-485-5p	0.709	0.3565679	0.87	0.219602	1.22708	0.52562
miR-545	33.1583	25.8879074	21.921333	13.22684	0.66111	0.52838
miR-203	0.785	0.30696145	0.9613333	0.390826	1.22463	0.53071
miR-28	1.331	0.5907972	1.097	0.086029	0.82419	0.5356
miR-340	1.25175	0.69231899	0.968	0.294455	0.77332	0.54173
miR-219	1.5865	0.90687357	2.2463333	1.78068	1.41591	0.54385
miR-23b	1.4185	0.76448392	1.1143333	0.318616	0.78557	0.55234
miR-433	0.64	0.29562138	0.77	0.222594	1.20313	0.55442
miR-423	1.3055	0.43233128	1.0913333	0.485768	0.83595	0.56423
miR-29c	1.20925	0.32280167	0.9693333	0.703432	0.8016	0.56515
miR-296	2.9475	2.36493897	2.09	0.911451	0.70908	0.58418
miR-101	1.10225	0.1627009	1.263	0.53764	1.14584	0.58682
miR-133b	0.598	0.34704178	0.73	0.202778	1.22074	0.5869
miR-339	0.9965	0.3608587	0.832	0.387106	0.83492	0.5873
miR-140	1.05325	0.22716129	0.9526667	0.292428	0.9045	0.62793
miR-519c	1.529	0.89591555	1.9353333	1.208048	1.26575	0.62822
miR-30c	0.863	0.10906267	0.8176667	0.126508	0.94747	0.63168
miR-186	1.0215	0.29924405	0.9163333	0.226102	0.89705	0.63465
miR-199a	0.9945	0.23368426	0.8686667	0.437008	0.87347	0.63917
miR-200a	1.64125	1.28107335	2.0416667	0.558299	1.24397	0.63977
miR-153	0.76275	0.33855317	0.6466667	0.255817	0.84781	0.64273
miR-100	1.84875	1.26723041	1.4526667	0.774562	0.78576	0.65633
miR-30a-3p	1.0205	0.2201613	1.1273333	0.383719	1.10469	0.65708
miR-139	0.61825	0.28670935	0.535	0.107014	0.86535	0.65848
miR-1	0.78675	0.22179476	0.899	0.431355	1.14268	0.66761
RNU6B	0.79325	0.22940703	0.8843333	0.310105	1.11482	0.67114
miR-425	0.89625	0.1075248	0.987	0.411955	1.10126	0.68211
miR-99a	1.80375	1.18254144	1.479	0.574062	0.81996	0.68406
miR-27b	1.42825	0.76288679	1.1733333	0.798839	0.82152	0.68558
miR-22	1.1615	0.37280692	1.2933333	0.445	1.1135	0.68642
miR-93	1.4515	0.58159407	1.6226667	0.468677	1.11792	0.69495
miR-449b	0.7745	0.48028082	0.6403333	0.318889	0.82677	0.69527
miR-594	0.59975	0.49227524	0.4316667	0.584283	0.71974	0.69572
miR-222	0.65075	0.28553736	0.7283333	0.204913	1.11922	0.70826
miR-422a	2.69025	1.44199315	2.301	1.008503	0.85531	0.70828
miR-15a	1.7	0.96465849	1.99	0.963894	1.17059	0.71001
miR-223	0.96825	0.33342003	1.0833333	0.45026	1.11886	0.71125
miR-565	0.4115	0.40022785	0.3236667	0.150268	0.78655	0.73731
miR-484	0.9815	0.19944841	1.0543333	0.350449	1.07421	0.73851
miR-148b	1.3395	0.40767348	1.2266667	0.43443	0.91576	0.73853
miR-128b	0.75325	0.46996906	0.643	0.398903	0.85363	0.75769
miR-299-5p	0.64375	0.33351899	0.7116667	0.141797	1.1055	0.75822
miR-330	2.56475	2.02394209	2.2183333	0.324149	0.86493	0.78572
miR-379	0.66975	0.29282119	0.722	0.120715	1.07801	0.78645
miR-338	1.464	0.80232454	1.662	1.111665	1.13525	0.7934
miR-125b	0.99475	0.20500305	0.952	0.203295	0.95702	0.79508
miR-17-5p	1.28225	0.47240616	1.4143333	0.871828	1.10301	0.80426
miR-200c	1.80575	1.19888792	1.5956667	1.043266	0.88366	0.81879
miR-192	0.84825	0.13459167	0.8053333	0.331627	0.94941	0.81993
miR-17-3p	1.76825	1.53401638	2.1	2.218274	1.18761	0.82261
miR-26a	1.4885	0.78630338	1.3603333	0.648908	0.9139	0.82832
miR-189	0.72575	0.41337503	0.7873333	0.25725	1.08485	0.83126
miR-572	0.507	0.34454801	0.459	0.244841	0.90533	0.84663
miR-27a	1.26775	0.75204183	1.178	0.408082	0.92921	0.86092
miR-184	1.6185	0.52238268	1.5566667	0.286741	0.9618	0.8623
miR-18a	1.38025	0.52023352	1.2856667	0.904826	0.93147	0.8665
miR-488	0.768	0.2062749	0.7146667	0.619691	0.93056	0.87542
miR-181d	1.2145	0.44892872	1.259	0.340762	1.03664	0.89231
let-7b	1.49275	0.88347057	1.4136667	0.683664	0.94702	0.9032
miR-380-5p	0.667	0.29559544	0.6453333	0.055185	0.96752	0.90729
miR-92	1.81475	1.01158271	1.7296667	1.051114	0.95312	0.91789
miR-210	1.25425	0.88605582	1.3093333	0.073759	1.04392	0.92058
miR-345	1.26725	0.54572116	1.225	0.536005	0.96666	0.92265
miR-518f	0.9345	0.46134766	0.9636667	0.41921	1.03121	0.93494
miR-142-3p	1.6355	0.81751555	1.6776667	0.182006	1.02578	0.93497
miR-491	0.6615	0.32211851	0.6753333	0.154746	1.02091	0.94874
miR-518d	1.545	1.4022123	1.5996667	0.232169	1.03538	0.95046
miR-214	1.082	0.16332993	1.1023333	0.69285	1.01879	0.95571
miR-151	1.45825	0.79300877	1.4896667	0.75322	1.02154	0.95985
miR-152	1.3565	0.8254738	1.3286667	0.448393	0.97948	0.96047
miR-597	0.81	0.16204526	0.8166667	0.208534	1.00823	0.96362
miR-410	0.66475	0.2446976	0.6683333	0.156596	1.00539	0.98335
miR-550	1.5525	0.83919823	1.5643333	0.561507	1.00762	0.98412
miR-502	1.67725	1.07493577	1.6833333	0.554203	1.00363	0.99331
miR-181b	1.35875	0.69139732	1.356	0.711981	0.99798	0.99609

### Disruption of Production of Mature miR-650 in AD Cases

MiRNAs are generated from long primary miRNAs and processed into precursor miRNAs, and finally formed mature miRNAs (15–18). Deregulated processing of miRNA, as one of the mechanisms to regulate miRNAs expression, has been observed in multiple diseases [17, 20]. We hypothesized that the increased miR-650 expression observed in AD cases might be due to an alteration in miRNA processing. Mature form of miR-650 was generated from the 5-prime strand of its precursor (Supplemental Fig. 1). To test this hypothesis, we analyzed the expression level of primary transcripts, precursor transcripts, and the mature form of miR-650 by quantitative real-time PCR. Surprisingly, we found that in contrast to the increased mature form of miR-650, levels of primary and precursor forms of miR-650 were significantly decreased in AD cases compared to controls (Fig. 1B–D). These results indicate that the processing of primary miR-650 to mature miR-650 are altered in AD patients.

### Bioinformatic Prediction of miR-650 Targets Related to AD Pathogenesis

We next performed bioinformatic analyses to identify the potential targets for the increased miRNAs. Utilizing commercially available software programs TargetScan, we found miR-650, which was significantly increased in AD samples, was predicted to bind the 3′ untranslated regions (3′-UTR) of Apolipoprotein E (APOE), Presenilin 1 (PSEN1), and Cyclin-Dependent Kinase 5 (CDK5) (Supplemental Fig. 2).

### miR-650 Is Able to Regulate Multiple Genes Implicated in AD

**Fig. 2:**
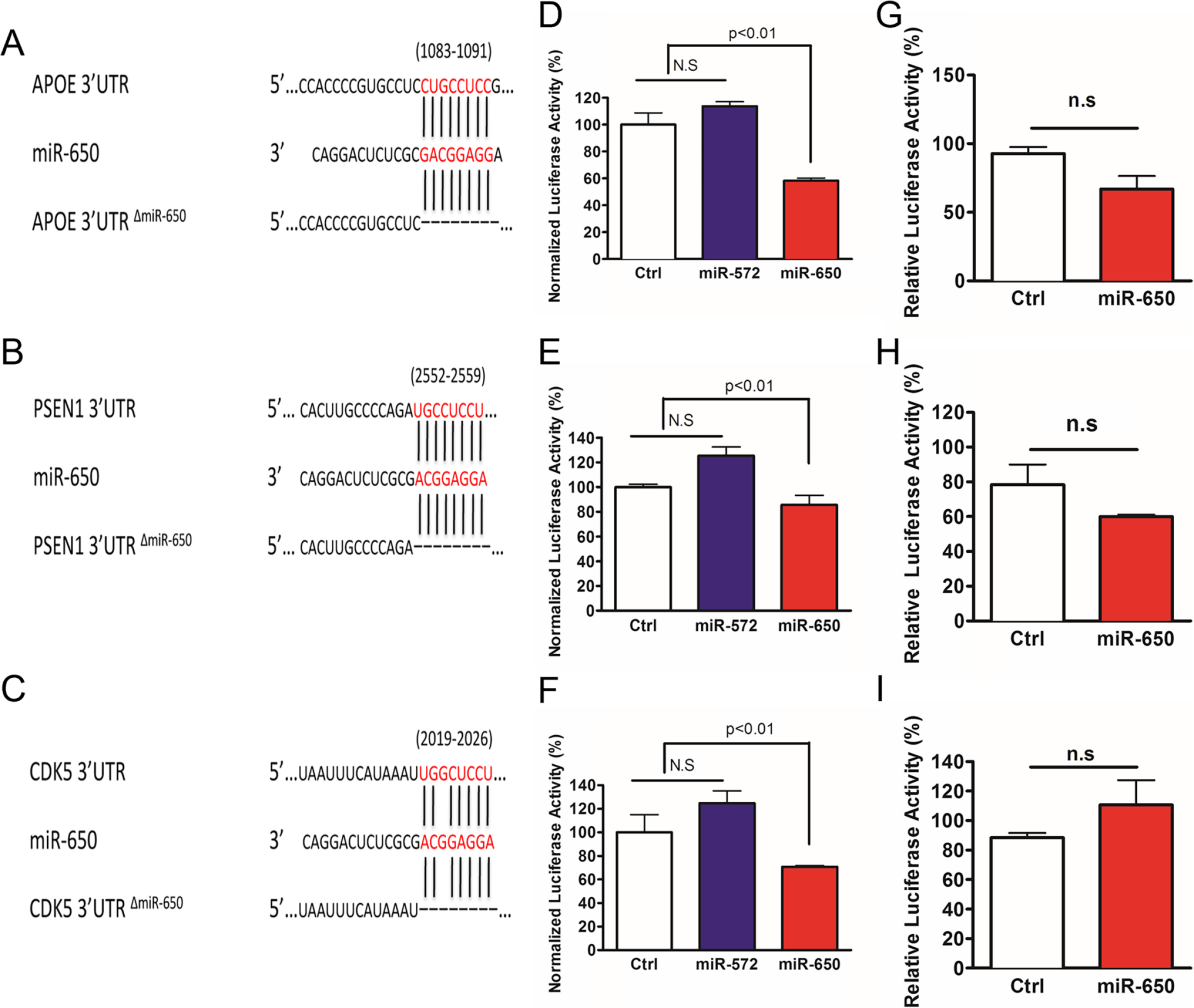
Expression with miR-650 significantly decreases expression of APOE, PSEN1, and CDK5 in vitro. **A**–**C** Predicted target alignment sequence for miR-650 with APOE, PSEN1, and CDK5 3′UTR. Also depicted is mutant 3′UTR vectors with deletion of miR-650 binding site sequence. **D**–**F** Expression of miR-650 leads to decreased expression of APOE (**D**), PSEN1 (**E**), and CDK5 (**F**) as assessed by a luciferase reporter. miR-572 was used as a negative control as it is not predicted to target any of these genes. **G**–**I** Deletion of miR-650 target sequence abolishes the significant finding of APOE (**G**), PSEN 1 (**H**), and CDK5 (**I**). Values are mean ± SD from three independent experiments; each experiment included triplicate for every co-transfection. One-way ANOVA, *p* < 0.01, n.s., no significant

The prominent function of miRNA is to post-transcriptionally regulate the expression target genes [17, 19, 20]. To further study the function of miR-650, we cloned the 3′-UTR of *APOE*, *PSEN1*, and *CDK5* into a luciferase vector and co-transfected each with miR-650 into HEK293 cells. Target alignment sequence of each 3′UTR to miR-650 is shown in Fig. 2A–C. To avoid non-specific effects, we used miR-572 as a negative control, as it is not predicted to target any of these three genes. The cells were collected 48 h post-transfection, and the luciferase assay was performed. Compared to control, miR-650 was able to robustly decrease luciferase activity (Fig. 2D–F). These results indicate miR-650 can bind to the 3′UTR of the predicted targets *APOE*, *PSEN1*, and *CDK5*.

To further validate these results, we constructed mutant 3′-UTR-luciferase vectors by deleting the binding sites of miR-650 in the 3′-UTRs of the predicted targets (Fig. 2A–C). Cotransfection and luciferase assays were performed as described above, and, as expected, miR-650 was no longer able to significantly decrease the luciferase activity (Fig. 2G–I). Together, these data indicate that miR-650 is able to specifically regulate the expression of *APOE*, *PSEN1*, and *CDK5*.

### Overexpression of miR-650 Downregulates CDK5 and Ameliorates AD Pathogenesis in APP/PSEN1 Transgenic Mice

**Fig. 3:**
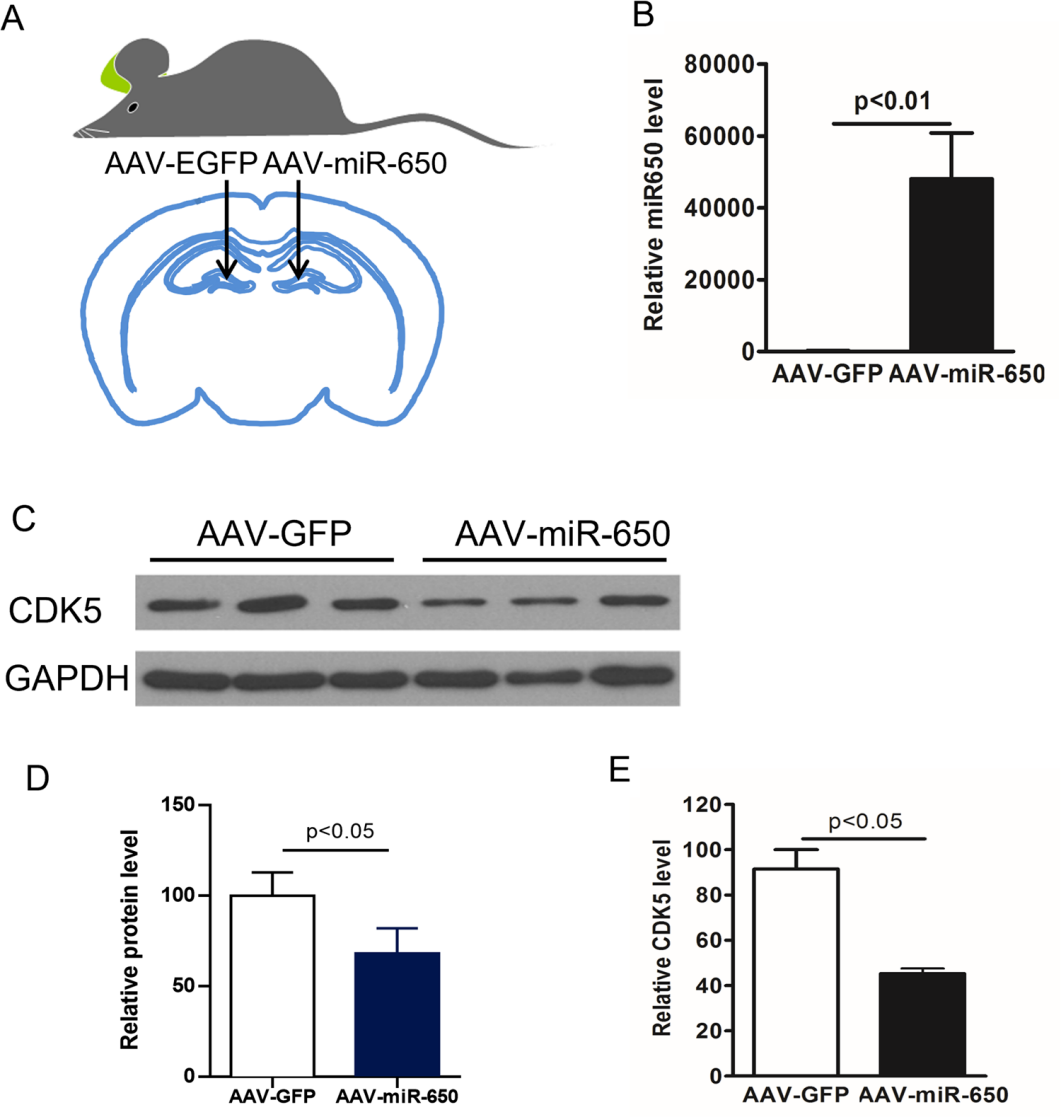
Overexpression of miR-650 decreases CDK5 levels in vivo. **A** The dentate gyrus (DG) of APP-PSEN1 mice was injected bilaterally with adeno-associated virus (AAV) vectors: AAV-GFP control virus was injected in the left DG and AAV-miR-650 virus in the right DG (*n* = 3). **B** Expression of miR-650 was only seen in the hippocampus sections that were injected with AAV-miR-650 (unpaired *t*-test, *p* < 0.01). **C**–**D** Western blot analysis shows a significant decrease in CDK5 levels on hippocampus samples injected with AAV-miR-650 (unpaired *t*-test, *p* < 0.01). **E** Transcript levels of CDK5 were also significantly decreased in the samples injected with AAV-miR-650 (unpaired *t*-test, *p* < 0.01)

We next investigated whether overexpression of miR-650 could modulate the expression of the predicted target *CDK5* in vivo. To perform this experiment, we used APP/PSEN1 double transgenic mice and measured CDK5 levels. APP/PSEN1 double transgenic mice contain mutant forms of APP and PSEN1 that are associated with early onset AD [42]. Virus injections were performed bilaterally on the hippocampus of each mouse (*n* = 5): injection was administered at the left lateral DG for AAV-GFP and the right lateral DG for AAV-miR-650 (Fig. 3A). One month after virus grafting, the mice were sacrificed, and hippocampi were dissected out. To confirm the efficiency of virus injection and infection, we first performed western blot to analyze the expression of GFP. We found both AAV-GFP and AAV-GFP-miR-650 grafted samples displayed equal levels of expression of GFP, confirming the efficiency of the injection and virus infection in vivo (Supplemental Fig. 3); importantly, miR-650 expression was only seen with the AAV-miR-650 samples (Fig. 3B). These samples were also used to measure CDK5 levels, and we found that CDK5 protein (Fig. 3C–D) and transcript levels (Fig. 3E) was significantly decreased by over-expressing miR-650. These data indicated that miR-650 can regulate CDK5 protein levels in vivo. Taken together, these data further confirmed CDK5 is a bona fide target of miR-650 in vivo.

**Fig. 4:**
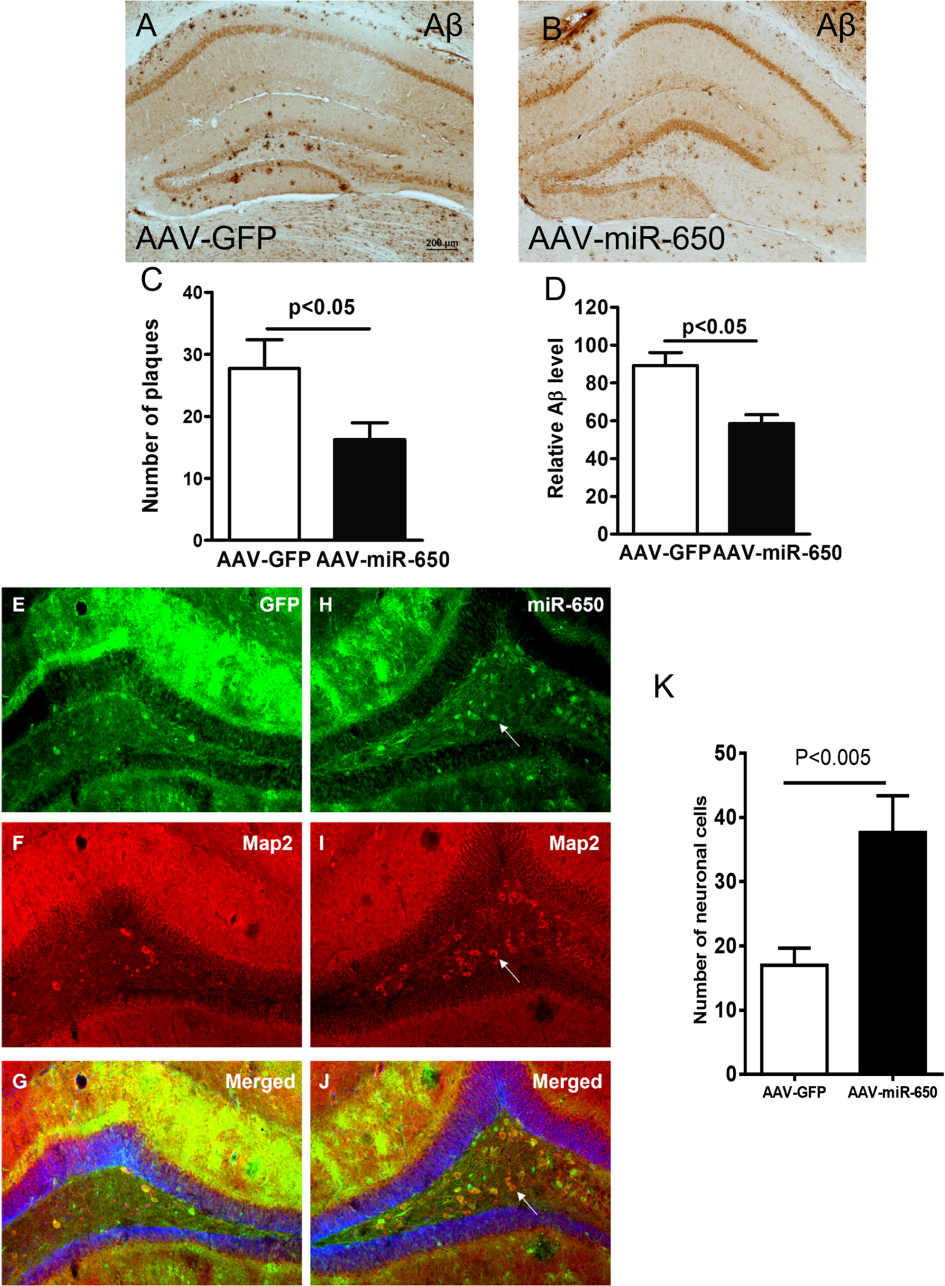
Overexpression of miR-650 ameliorates AD pathogenesis in APP/PSEN1 transgenic mice. **A**–**B** Immunohistochemistry of beta-amyloid for hippocampal sections from AAV-GFP control mice (**A**) and AAV-miR-650 mice (**B**). **C** AAV-miR-650 brain sections show significantly fewer number of plaques. **D** AAV-miR-650 mice show significantly lower amyloid-β (Aβ) levels (*n* = 3, unpaired *t*-test, *p* < 0.05). **E**–**J** Immunostaining of hippocampal sections for AAV-GFP control mice (**E**–**G**) and AAV-miR-650 mice (**H**–**J**). Neurons are indicated by red staining with MAP2 antibody. Arrow indicates increased neuronal staining in the presence of miR-650 overexpression. **K** AAV-miR-650 mice show significantly increased number of neuronal cells (*n* = 3, unpaired *t*-test, *p* < 0.05)

To determine if reduced CDK5 induced by miR-650 overexpression has neuronal effects, we performed immunostaining of neuronal marker microtubule-associated protein 2 (MAP2) with sections from virus injected APP/PSEN1 mice [43]. First, the staining revealed that in the presence of overexpressed miR-650 the number of plaques and Aβ levels were significantly reduced (Fig. 4A–D). Furthermore, the overexpression of miR-650 also significantly increased MAP2 positive cells (neuronal cells) in the dentate gyrus of APP/PSEN1 mice, compared to GFP virus injection (Fig. 4E–K). These results suggest miR-650 has the ability to ameliorate neuronal deficits in APP mice.

## Discussion

In this study, we described a novel miR-650-CDK5 regulatory axis that is involved in the physiopathology of AD. Significantly increased miR-650 was found in sporadic AD patients. Furthermore, in vitro assay demonstrated that *APOE*, *PSEN1*, and *CDK5* are all specifically targeted by miR-650*.* Importantly, CDK5 expression was shown to be regulated by miR-650 in vivo using a mouse model for early onset AD: overexpression of miR-650 significantly inhibited CDK5 expression and increased the immunostaining intensity of neuronal cells in APP/PSEN1 mice.

Irregular expression of miR-650 has been well documented in multiple types of human cancer. Several targets of miRNA-650 have been identified, including *ING4*, *CDK1*, *AKT2*, *EBF3*, and other factors [44–55]. Nonetheless, the importance of miR-650 in AD pathogenesis has not been defined. In our study, we observed a significant increase of miR-650 in AD patients. Predicted by bioinformatic analysis that CDK5 is one of the targets of miR-650, the direct interaction of miR-650 with CDK5 3′-UTR has been validated by in vitro luciferase functional studies using normal and mutated CDK5 3′-UTR structures. Furthermore, an inverse relationship between the expression of miR-650 and CDK5 was demonstrated by in vivo overexpression of miR-650 by adeno-associated virus leading to reduced CDK5 expression. Thus, our data suggested an important molecular link between miR-650 and CDK5 in AD mouse model.

CDK5 is an atypical proline-guided serine/threonine kinase that phosphorylates various substrate and exerts multiple regulatory roles in neuronal cells, as well as other cell types [56–58]. CDK5 is required for normal brain development, as evidenced by Cdk5-null mice not being viable past postnatal day zero [59]. The CDK5/p25 complex hyperphosphorylates Tau and leads to irreversible cytoskeletal disruption of neuronal cells and induced neuronal cell death [60]. Previous studies have demonstrated that CDK5 activity is increased in several different neurodegenerative diseases including AD, animal models of amyotrophic lateral sclerosis (ALS) and Niemann Pick type C (NPC) disease [61–63]. By manipulating CDK5 expression via overexpression of miR-650 in an AD mouse model, we showed that reduced plaques and Aβ presence in the brain. Thus, the miR-650-CDK5 regulatory axis defined in our study has important implications in AD pathogenesis.

Recent progress in the understanding of AD pathogenesis indicated that dysregulation of miRNA biogenesis was involved in neurodegeneration. On this regard, new methodological advances of AD therapy have developed short and synthetic antisense oligonucleotides (ASOs), that recognize target mRNA for posttranscriptional regulation to correct protein expression errors. The miRNA based ASOs design provided a possibility for synchronous regulation of protein expression and related signaling pathways [64, 65]. Thus, our finding about miR-650 involved in AD pathogenesis provides a potential target to develop ASOs therapeutics for AD.

In summary, our present results showed that miR-650 can regulate CDK5 both in vitro and in vivo. Over-expression of miR-650 can also significantly ameliorate neuronal deficits. These results suggest miR-650 is actively involved in neurodegeneration through modulating CDK5.

## Supplementary Information

Below is the link to the electronic supplementary material.

ESM 1 (PNG 338 kb)

High resolution image (TIF 710 kb)

ESM 2 (PNG 1217 kb)

High resolution image (TIF 2510 kb)

ESM 3 (PNG 113 kb)

High resolution image (TIF 230 kb)

## Data Availability

All data generated or analyzed during this study are included in this published article and its supplementary information files.
