# Supplementary figures and images for: MicroRNA-650 Regulates the Pathogenesis of Alzheimer’s Disease Through Targeting Cyclin-Dependent Kinase 5
Source: Mol Neurobiol. 2023 Jan 19;60(5):2426–41. doi: 10.1007/s12035-023-03224-y (PMC10039829; doi:10.1007/s12035-023-03224-y)

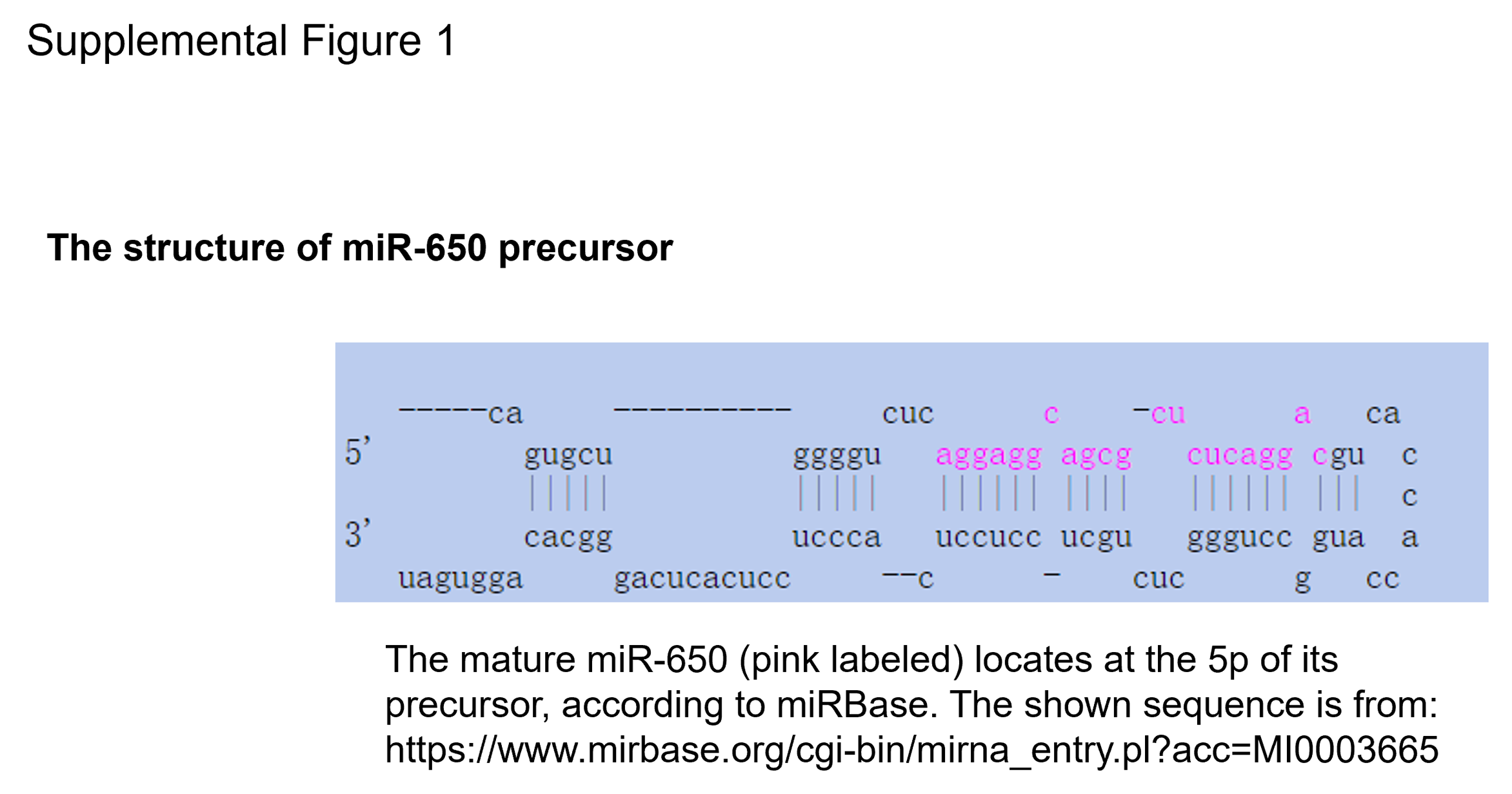

Supplement: Supplementary file 1 — (PNG 338 kb) [file 12035_2023_3224_Fig5_ESM.png]

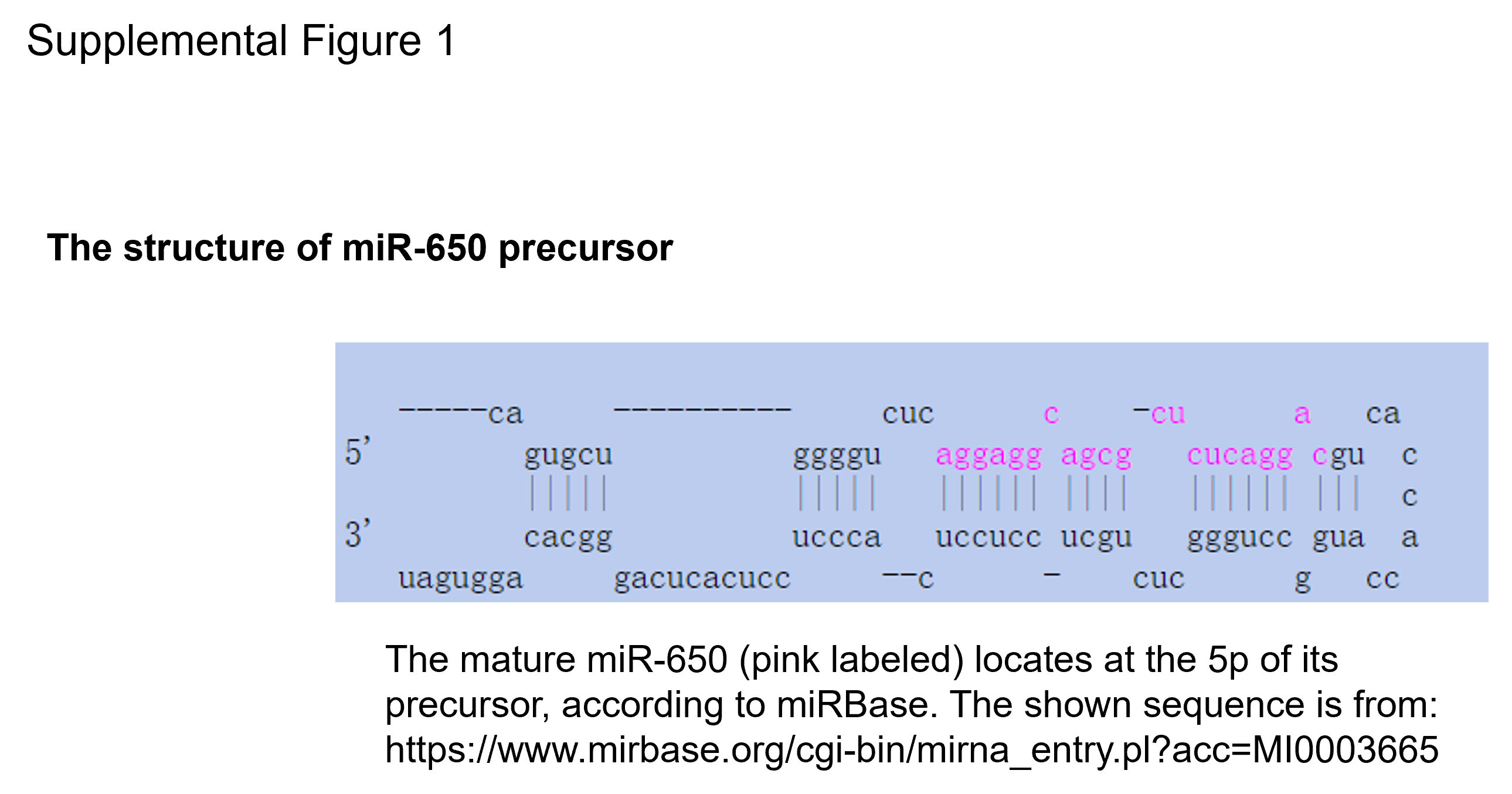

Supplement: Supplementary file 2 — High resolution image (TIF 710 kb) [file 12035_2023_3224_MOESM1_ESM.tif]

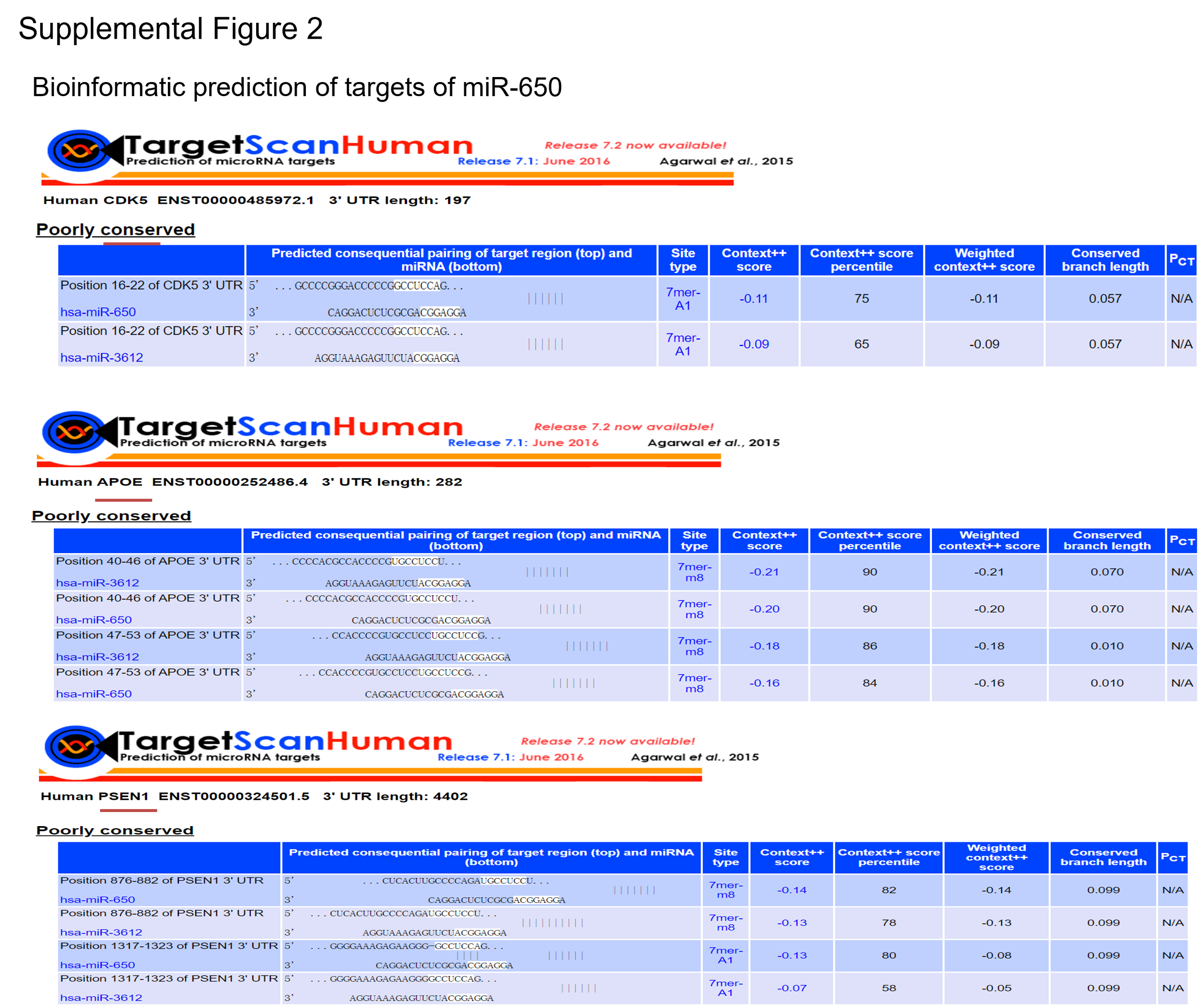

Supplement: Supplementary file 3 — (PNG 1217 kb) [file 12035_2023_3224_Fig6_ESM.png]

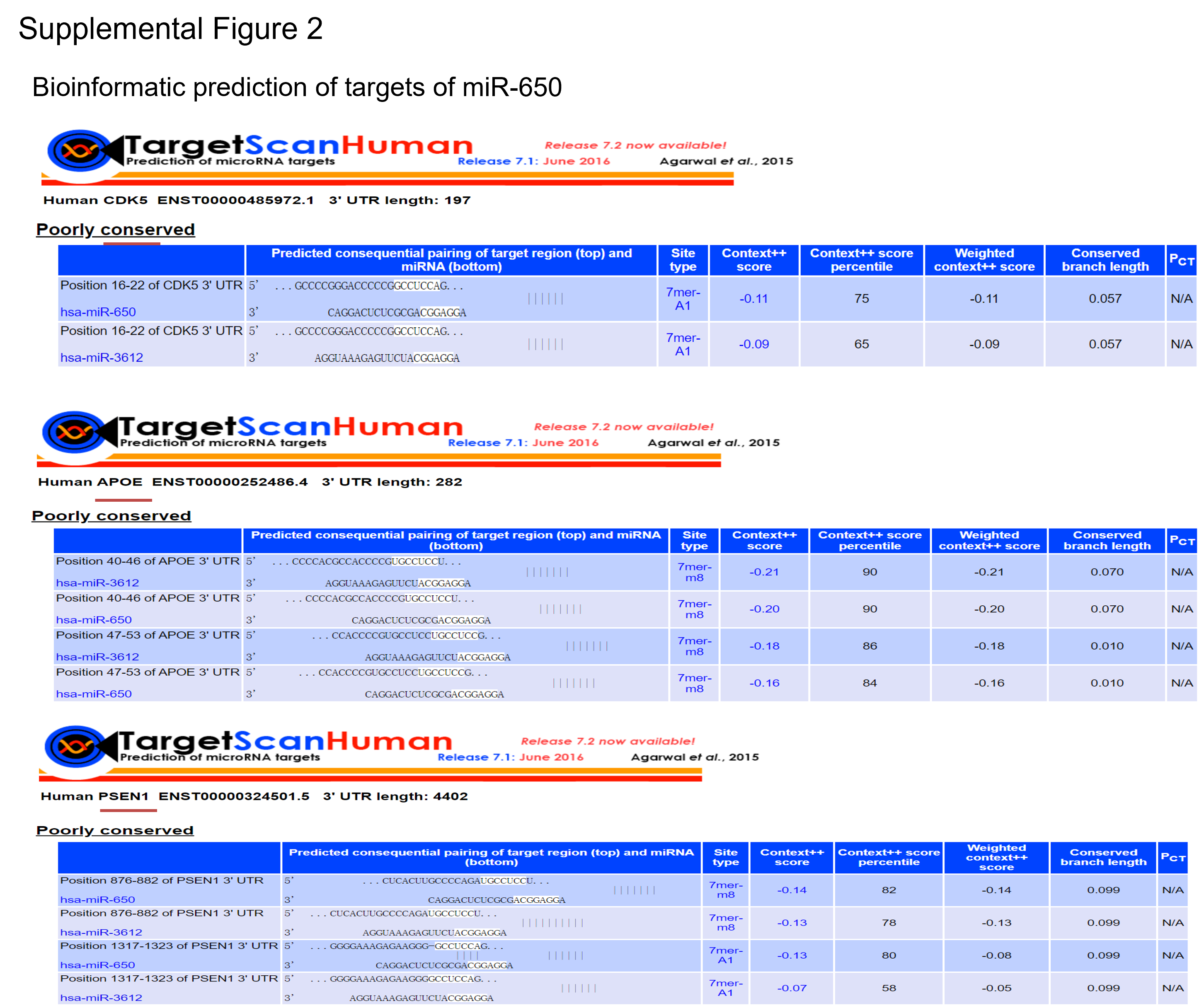

Supplement: Supplementary file 4 — High resolution image (TIF 2510 kb) [file 12035_2023_3224_MOESM2_ESM.tif]

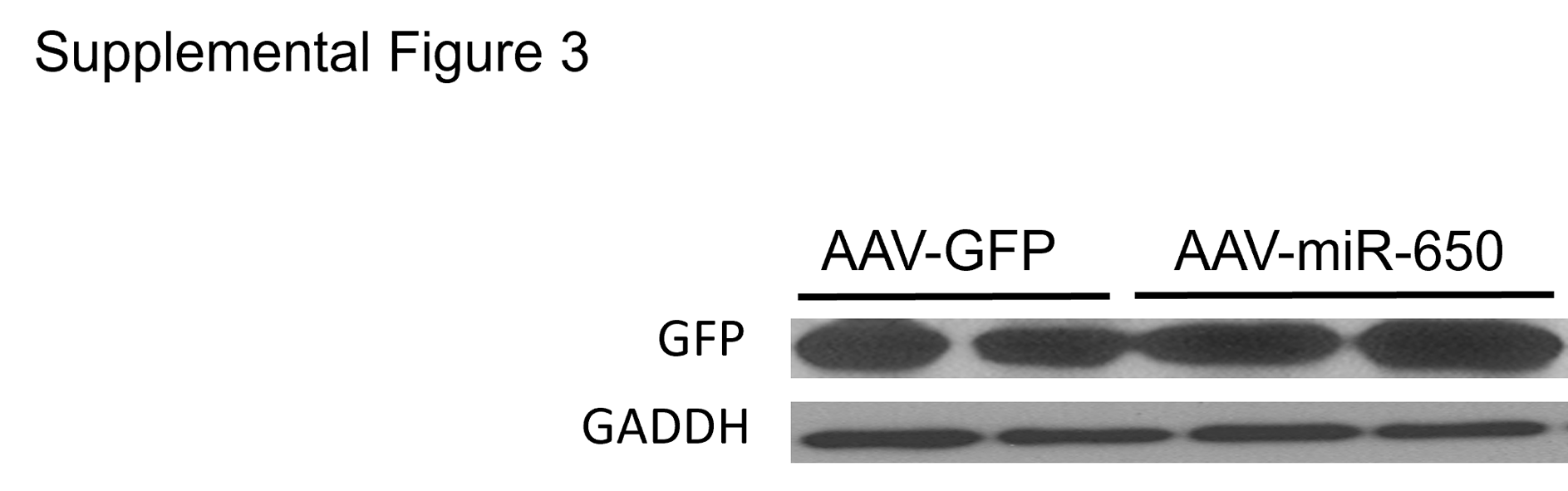

Supplement: Supplementary file 5 — (PNG 113 kb) [file 12035_2023_3224_Fig7_ESM.png]

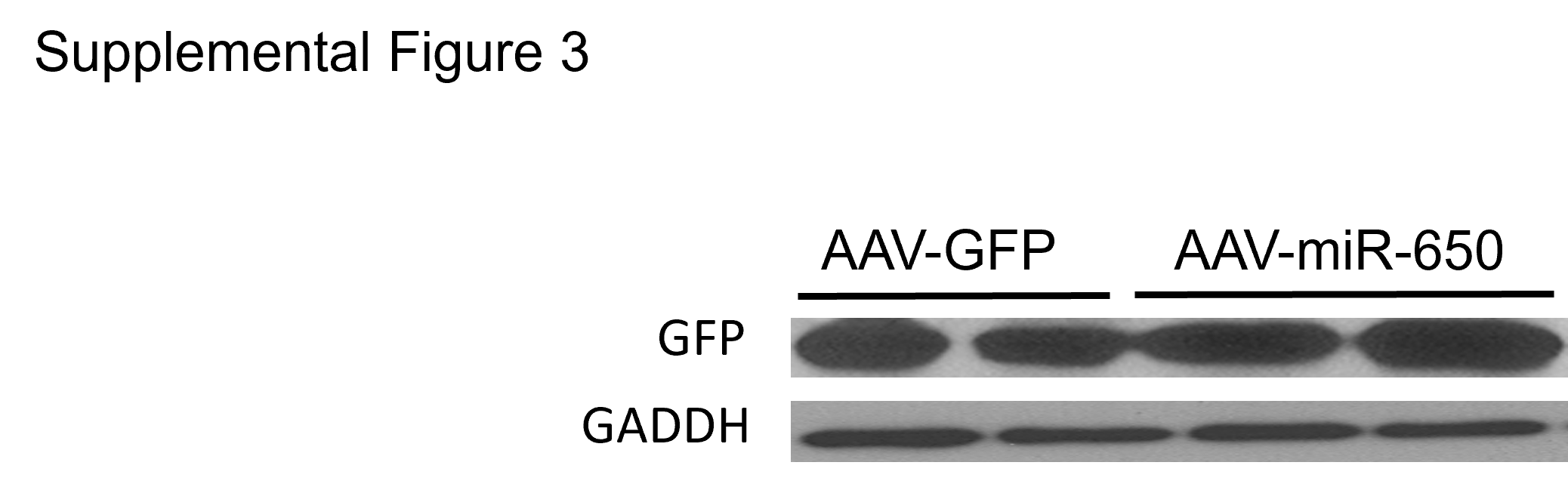

Supplement: Supplementary file 6 — High resolution image (TIF 230 kb) [file 12035_2023_3224_MOESM3_ESM.tif]
